# Structure–function relationship of dynorphin B variants using naturally occurring amino acid substitutions

**DOI:** 10.3389/fphar.2024.1484730

**Published:** 2024-10-30

**Authors:** Luca Zangrandi, Barbara Fogli, Anna Mutti, René Staritzbichler, Victoria Most, Peter W. Hildebrand, Regine Heilbronn, Christoph Schwarzer

**Affiliations:** ^1^ Institute of Pharmacology, Medical University of Innsbruck, Innsbruck, Austria; ^2^ Clinic for Neurology and Experimental Neurology, AG Gene Therapy, Charité - Universitätsmedizin Berlin, corporate member of Freie Universität Berlin and Humboldt-Universität zu Berlin, Berlin, Germany; ^3^ Institute of Medical Physics and Biophysics, University of Leipzig, Leipzig, Germany; ^4^ Charité – Universitätsmedizin Berlin, corporate member of Freie Universität Berlin and Humboldt-Universität zu Berlin, Institute of Medical Physics and Biophysics, Berlin, Germany

**Keywords:** opioid receptors, epilepsy, functional selectivity, G-protein-coupled receptors, selectivity

## Abstract

Dynorphins (Dyn) represent the subset of endogenous opioid peptides with the highest binding affinity to kappa opioid receptors (KOPrs). Activation of the G-protein-coupled pathway of KOPrs has strong anticonvulsant effects. Dyn also bind to mu (MOPrs) and delta opioid receptors (DOPrs) with lower affinity and can activate the β-arrestin pathway. To fully exploit the therapeutic potential of dynorphins and reduce potential unwanted effects, increased selectivity for KOPrs combined with reduced activation of the mTOR complex would be favorable. Therefore, we investigated a series of dynorphin B (DynB) variants, substituted in one or two positions with naturally occurring amino acids for differential opioid receptor activation, applying competitive radio binding assays, GTPγS assays, PRESTO-Tango, and Western blotting on single-opioid receptor-expressing cells. Seven DynB derivatives displayed at least 10-fold increased selectivity for KOPrs over either MOPrs or DOPrs. The highest selectivity for KOPrs over MOPrs was obtained with DynB_G3M/Q8H, and the highest selectivity for KOPrs over DOPrs was obtained with DynB_L5S. Increased selectivity for KOPr over MOPr and DOPr was based on a loss of affinity or potency at MOPr and DOPr rather than a higher affinity or potency at KOPr. This suggests that the investigated amino acid exchanges in positions 3, 5, and 8 are of higher importance for binding and activation of MOPr or DOPr than of KOPr. In tests for signal transduction using the GTPγS assay, none of the DynB derivatives displayed increased potency. The three tested variants with substitutions of glycine to methionine in position 3 displayed reduced efficacy and are, therefore, considered partial agonists. The two most promising activating candidates were further investigated for functional selectivity between the G-protein and the β-arrestin pathway, as well as for activation of mTOR. No difference was detected in the respective read-outs, compared to wild-type DynB. Our data indicate that the assessment of affinity to KOPr alone is not sufficient to predict either potency or efficacy of peptidergic agonists on KOPr. Further assessment of downstream pathways is required to allow more reliable predictions of *in vivo* effects.

## Introduction

The kappa opioid receptor (KOPr) is one of the three classical opioid receptors. All three, KOPr, mu opioid receptor (MOPr), and delta opioid receptor (DOPr), belong to the class of 7-transmembrane receptors and couple to G_i/o_ proteins. KOPrs were discussed as an antinociceptive drug target already over 30 years ago. However, dysphoric side effects (Barber and Gottschlich, 1997) stopped further drug development. KOPrs typically signal through G-protein-coupled and β-arrestin-dependent pathways (Bruchas and Chavkin, 2010). Some studies suggested that antinociceptive and anticonvulsant effects depend on G-protein signaling, while aversive effects depend on GRK-3/β-arrestin recruitment and subsequent phosphorylation of p38 MAPK (Bruchas et al., 2007; Ehrich et al., 2015). Others suggested that aversion might be induced independent of β-arrestin recruitment (White et al., 2015). Phosphoproteomic analyses revealed that the activation of mTOR is involved in the aversive effects of KOPr activation (Liu et al., 2018; Liu et al., 2019).

A few years earlier, functional selective agonists inducing antinociceptive and anticonvulsant effects lacking aversive effects were discovered (Lindesay et al., 1989; Raehal and Bohn, 2014; White et al., 2014; Liu et al., 2018). Presently available biased agonists are small-molecule drugs with a relatively short half-life. These are well-suited for controllable, short-term treatments such as for post-surgical pain. Due to their apparent lack of abuse potential KOPr agonists could be used to replace MOPr agonists, which are seen as increasingly problematic in light of the ongoing opioid crisis. Much emphasis is placed on the development of G-protein biased agonists with a better profile of side effects (Kaski et al., 2021).

Diseases like focal epilepsies, characterized by sporadic, unpredictable seizures, require targeted “drug on demand” therapies. This can be achieved by gene therapy. The activation of KOPrs by prodynorphin (pDyn)-derived peptides has been implicated to control seizures in epilepsy. To achieve targeted and lasting peptide delivery, we have developed an AAV vector-based gene therapy, delivering pDyn to the epileptogenic focus. In animal models of the disease, expression of dynorphins (Dyn) and their release on demand led to long-term suppression of seizure development (Agostinho et al., 2019). To exploit the full potential of Dyn, higher selectivity for KOPr compared to MOPr or DOPr might be achieved by modified Dyn peptides. For gene therapy, such modifications are restricted to substitutions with naturally occurring amino acids. Increased selectivity for KOPr activation may boost the relative efficacy of gene therapy for epilepsy since parallel activation of MOPr and/or DOPr is considered pro-convulsant (Burtscher and Schwarzer, 2017). Furthermore, functional selectivity for the G-protein pathway rather than the β-arrestin pathway would be considered beneficial (Zangrandi et al., 2016).

Native human pDyn is processed to seven neuropeptides. They display distinct properties in terms of the binding, selectivity, and internalization of KOPr. All of them have the highest affinity for the KOPr, but also bind to MOPr and DOPr with lower potency. In the hippocampus, striatum, and cortex, dynorphin B (DynB) and alpha-neoendorphin (α-Neo) are predominant, while dynorphin A (DynA) is the major product in the spinal cord [for review, see Schwarzer (2009) and Chavkin (2013)]. DynA (1–13), DynB (1–11), and α-Neo display similar binding affinities and comparable efficacies to stimulate the G-protein at the human KOPr. While the binding selectivity for KOPr over MOPr and DOPr is comparable for the main peptides (Chen et al., 2007), the selectivity for the activation of the G-protein appears to be lower for α-neo (Fricker et al., 2020).

There are several studies on modified DynA affinity and selectivity, which occurs mostly through chemical modification of single amino acids or through introduction of non-naturally occurring amino acids (Aldrich et al., 2001; Vig et al., 2003; Fang et al., 2009; Lohman et al., 2023). For *in vivo* gene therapy, only natural amino acids can be incorporated in newly synthesized peptides.

The aim of this study was to investigate whether natural amino acid substitutions in DynB improve the efficacy and selectivity for KOPr activation. DynB is one of the two major pDyn-derived products in the hippocampus and cortex, key sites in different types of epilepsy. In addition, DynB is known to have an inherently higher functional selectivity for the G-protein pathway than α-Neo (Fricker et al., 2020). A previous alanine scan study for DynB (Joshi et al., 2017) suggested alterations in positions 3 and 10 of DynB as the best candidates to improve the selectivity without reducing the affinity to KOPr. However, Joshi et al. focused on the binding affinity only. Here, we explored several alternative amino acid exchanges and went beyond receptor binding to also study the activation of the G-protein as a functional readout. In addition, recruitment of β-arrestin and the functional selectivity for downstream pathways were investigated for selected peptides.

## Material and methods

### Drugs and antibodies

Wild-type and mutated peptides were purchased from GenScript Biotech Corporation (Piscataway, NJ, United States). [^3^H]-DAMGO, [^3^H]-diprenorphine, [^3^H]-U-69,593, and [^35^S]-GTPγS were purchased from PerkinElmer (Waltham, MA, United States). DAMGO, GTPγS, and U-69593 were purchased from Sigma-Aldrich (Vienna, Austria). Diprenorphine was obtained from Tocris Bioscience (Abingdon, United Kingdom). Antibodies for Western blot were purchased from Cell Signaling Technology (Massachusetts, United States): ERK (#4,695), phosphor-ERK (#9106), p70 (#9202), phosphor-p70 (#9234), p38 (#9212), phosphor-p38 (#4511), and cofilin (#5175).

### Molecular biology

For PRESTO-Tango experiments, the human OPRK cDNA (DNASU Plasmid Repository HsCD00515607) (Seiler et al., 2014) was cloned into OPRK1-Tango, which was a gift from Bryan Roth (Addgene plasmid # 66462) (Kroeze et al., 2015). The TetOn-eGFP was a gift from Brad Zuchero [Addgene plasmid #89453; (Harterink et al., 2017)], and pCDNA3.1(+)-CMV-bArrestin2-TEV was a gift from Bryan Roth (Addgene plasmid #107245).

### Cell culture

CHO cells stably transfected with human KOPr, MOPr, and DOPr (hKOPr-CHO, hMOPr-CHO, and hDOPr-CHO, respectively) were provided by Prof. Liu-Chen (Temple University, Philadelphia, United States). The cells were grown in Gibco™ Dulbecco’s minimal essential medium (DMEM) for hKOPr-CHO or Gibco™ DMEM/F-12 for hMOPr and hDOPr. Both media were supplemented with 10% FBS (Gibco™), GlutaMAX (100 μg/mL; Gibco™), penicillin/streptomycin (100 μg/mL; Gibco™), and geneticin (G418, 400 μg/mL; Gibco™). HEK 293 cells were grown in high-glucose DMEM supplemented with 10% FBS (Gibco™), GlutaMAX (100 μg/mL; Gibco™), and penicillin/streptomycin (100 μg/mL; Gibco™). Cell cultures were maintained in a Heracell™ 150i CO_2_ incubator at 37°C and 5.0% CO_2_.

### Radio binding assay

Competitive heterologous binding assays were conducted on hKOPr-CHO, hMOPr-CHO, and hDOPr-CHO cell membranes. The membranes were prepared in 50 mM Tris-HCl buffer, pH 7.7. Cells were harvested by scraping the plates using a rubber policeman and then centrifuged at 500 *g* for 10 min. The cell pellet was resuspended in Tris-HCl, homogenized using a Dounce homogenizer, and centrifuged at 27,000 *g* for 15 min. The pellets were resuspended in Tris-HCl and homogenized through a 27G needle. The protein content was quantified using the ROTI^®^Quant universal kit (Carl Roth GmbH, Karlsruhe, Germany) The homogenate was stored at −70°C until use. Binding assays were conducted using [^3^H]-U69,593, [^3^H]-DAMGO, and [^3^H]-diprenorphine at a final concentration of 1 nM for labeling κ, μ, and δ opioid receptors, respectively. Non-specific binding was determined by using 1 μM of the unlabeled counterpart of each radioligand. Cell membranes (10–30 μg) were incubated with the appropriate radioligand, and increasing concentrations of the test peptide were diluted in 50 mM Tris-HCl, pH 7.4, in a total volume of 1 mL 50 mM Tris-HCl, pH 7.4 for 60 min at 25°C. Peptide solutions were replaced by a diluent for untreated controls. After incubation, reactions were terminated by rapidly washing three times with Tris-HCl and filtration through glass fiber filters (GF/C Whatman) on a Brandel M-24 Cell Harvester. The bound radioactivity was measured by liquid scintillation (Carl Roth GmbH, Karlsruhe, Germany) counting on a Packard 1600 TR Tri-Carb Beta counter (Canberra, Belgium).

### [^35^S]GTPγS functional assays for κ opioid receptors

Functional assays were conducted on hKOPr-CHO, hMOPr-CHO, and hDOPr-CHO cell membranes (see above). The membranes were prepared in a buffer containing 20 mM HEPES, 10 μM GDP, cell membrane (10–30 μg), and 0.05 nM [^35^S]GTPγS. The buffer containing the cell membranes was incubated with an increasing concentration of the test peptide diluted in 1 × HEPES, pH 7.4, in a total volume of 1 mL, for 60 min at 25°C. The peptide solutions were replaced by diluents for untreated controls. Non-specific binding was determined using unlabeled 10 μM GTPγS. Samples were rapidly washed three times with Tris-HCl and filtered through glass fiber filters (GF/B Whatman). The bound radioactivity was measured by liquid scintillation (Carl Roth GmbH, Karlsruhe, Germany) counting on a Packard 1600 TR Tri-Carb Beta counter (Canberra, Belgium).

### PRESTO-Tango

PRESTO-Tango assays (Kroeze et al., 2015; Lieb et al., 2021) were conducted on HEK 293 cells. On day 1, 20,000 cells/well were seeded in a clear-bottomed black 96-well plate. The following day (day 2), the cells were transfected using a 1:1:1:1 DNA ratio of tTA-hKOPr (receptor):TEV-β-arrestin:Tet-GFP:mCherry (100 ng*).* PEI MAX (Polysciences, Inc., PA, United States) diluted in Opti-MEM (Gibco-ThermoFisher) was used as a transfection agent at a PEI-to-DNA ratio of 2:1. On day 3, the transfected cells were treated overnight with the selected peptides properly diluted in DMEM. Peptide solutions were replaced by diluents for untreated controls. On day 4, the plates were read in a Spark Tecan plate reader at integration times of 1 s per well with excitation filters set to 485 ± 20 and 535 ± 25 nm and emission filters to 535 ± 25 and 595 ± 35 nm for GFP and mCherry, respectively. MCherry was measured to control for comparable cell numbers transfected.

The results in the form of relative fluorescence units (RFUs) were exported into Excel spreadsheets, and GraphPad Prism was used for analysis of data.

### Western blots

hKOPr-CHO cells were seeded in 10-cm dishes and cultured until they reached 70%–80% confluency. Overnight serum starvation was initiated by replacing the culture medium with FBS-free medium [DMEM supplemented with 100 μg/mL GlutaMAX, 100 μg/mL penicillin/streptomycin, and 400 μg/mL geneticin (G418)]. For the treatment, cells were incubated for 10 min at 37°C/5% CO_2_ with 1 µM DynA_WT, DynB_WT, DynB_L5S, or DynB_G3A/Q8A diluted in an FBS-free medium. The FBS-free medium was used as the control. After the treatment, cells were rinsed and harvested in cold PBS by scraping the dishes using a rubber policeman. Cell suspensions were centrifuged at 4°C for 7 min at 21,000 *g*, and the supernatants were removed. Pellets were then resuspended and washed in ice-cold PBS. The pellets were then resuspended in lysis buffer (RIPA 1 ×) containing phosphatase and protease inhibitors (Halt™ Protease and Phosphatase Inhibitor Cocktail, Thermo Scientific) and centrifuged at 4°C for 20 min at 16,000 g. Total proteins of the supernatant cell lysate were quantified by the biuret assay (Carl Roth GmbH, Karlsruhe, Germany). Antibody linearity ranges were determined for target proteins and the internal loading control, and the combined linear range was then used to determine how much sample should be loaded in order to produce a linear signal response. Thus, 10–20 μg of total lysate proteins was denatured in Laemmli sample buffer containing *β*-mercaptoethanol and heated for 2 min at 98°C for SDS-PAGE on pre-cast NuPAGE 4%–12% Bis–Tris gels (Invitrogen) or 10% Tris gels. Gels were run for 1 h at 180 V, and proteins were then electrophoretically transferred (250 mA) to polyvinylidene difluoride membranes (Hybond P; Amersham Biosciences, Little Chalfont, United Kingdom). The blots were blocked for 1 h in 5% dried skimmed milk or BSA in TBS-0.1% Tween 20 and incubated overnight at 4°C with phospho-antibodies or 1 h at RT with total proteins. The following antibody dilutions were used: ERK (Cell Signaling #4695; 1:4,000), phosphor-ERK (Cell Signaling #9106; 1:4,000), p70 (Cell Signaling #9202; 1:1,000), phosphor-p70 (Cell Signaling #9234; 1:1,000), p38 (Cell Signaling #9212; 1:1,000), phosphor-p38 (Cell Signaling #4511; 1:1,000), and cofilin (Cell Signaling #5175; 1:8,000). Immunoreactive bands were detected by incubating the membranes in a horseradish peroxidase-conjugated secondary antibody (1:5,000, Invitrogen), followed by incubation with enhanced chemiluminescence reagent (Merck Immobilon). Chemiluminescence was then visualized using the Fusion SL-4 Vilber Lourmat imaging system (Peqlab, Erlangen, Germany), and densitometric analysis was carried out using the ImageJ gel analyzer function. To avoid errors introduced by stripping the membranes, total and phosphorylated proteins were analyzed from parallel gels. All data were normalized to cofilin as a loading control. The results are given as the ratio of phosphorylated to non-phosphorylated proteins.

### Biased factor calculation

To perform biased factor analyses, we followed the step-by-step protocol described by Nagi and Pineyro (2016). In short, curve fitting was used to calculate τ and Ka values for G-protein and β-arrestin pathway activation. Log(τ/Ka) ratios were subtracted by log(τ/Ka) of DynA as the reference to yield ΔLog(τ/Ka) values for each peptide. In a final step for each peptide, ΔLog (τ/Ka) values for G-protein pathway activation were subtracted from ΔLog(τ/Ka) values for β-arrestin pathway activation and *vice versa* to calculate ΔΔLog(τ/Ka). The biased factor is yielded by delogging ΔΔLog (τ/Ka).

## Results

### Selection of DynB mutants

A total of 13 DynB amino acid exchange variants were selected for the screening of their pharmacological profile. DynB variants G3A, Q8A, K10A, G3A/Q8A, and G3A/K10A were selected according to previously published data showing that these alanine shifts did not affect the binding properties of endogenous DynA or DynB peptides (Naqvi et al., 1998; Joshi et al., 2017). One mutant (Y1F) was included to study the role of the hydroxyl groups on the tyrosine in position 1. The remaining DynB derivatives (G3M, L5S, Q8H, G3A/L5S, G3A/Q8H, G3M/L5S, and G3M/Q8H) were the results of *in silico* screening using Rosetta’s FlexPepDock docking approach (Raveh et al., 2010) and Rosetta’s fixbb design application (Kuhlman et al., 2003) in combination with classical molecular dynamics simulations to predict DynB variants with improved binding selectivity for KOPr compared to MOPr or DOPr (Hildebrand et al., 2019). Herein, simulations of the empty/unbound receptors were used to determine conformations accessible to the rather bulky peptidic ligands. Based on the conserved N-terminal YGGFL motif, spatial constraints for the docking were derived from simulations of receptor–peptide complexes. FlexPepDock translates these constraints into a pseudo-potential that guides the docking instead of enforcing the constraints. Variants were created at specific sequence positions for an ensemble of docking poses. An ensemble approach accounts for the dynamic nature of the systems within the static framework of protein design, in which the backbone is fixed. After energy minimization, mutations that were frequently found for KOPr and rarely for MOPr and, at the same time, showed an energetic improvement over MOPr were selected as candidates.

### Affinity and selectivity (radio binding assay)

Radio binding assay (RBA) experiments were conducted on the selected DynB variant peptides in order to assess their affinity for the canonical opioid receptors. The aim was to explore whether DynB variant selectivity for hKOPr is enhanced compared to the endogenous wild-type ligand.

Affinity levels of DynA and DynB for KOPr were measured within the expected range, with DynA affinity for hKOPr (Ki = 0.04 ± 0.0 nM) being 10-fold higher than that of DynB (Ki = 0.72 ± 0.18 nM). The selectivity of DynA for hKOPr over hMOPr and hDOPr (54-fold and 826-fold, respectively) was superior to that of DynB (16-fold over hMOPr and 112-fold over hDOPr; Table 1). These results are in line with previously published data (Schwarzer, 2009) and support the validity of our experimental settings.

**TABLE 1:** Binding affinities at the human opioid receptors.

	Ki [nM ± SEM(n)]			Selectivity	
	hKOPr	hMOPr	hDOPr	MOPr/KOPr	DOPr/KOPr
DynA_WT	0.04 ± 0.00 (12)	2.3 ± 0.5 (5)	35.5 ± 3.6 (8)	54	826
DynB_WT	0.72 ± 0.18 (6)	11.6 ± 2.6 (5)	80.2 ± 6.4 (7)	16	112
DynB_G3A	2.13 ± 0.75 (6)	82.3 ± 7.9 (5)	317 ± 34.1 (7)	39	149
DynB_Q8A	1.56 ± 0.29 (4)	21.9 ± 5.9 (4)	230 ± 30.3 (5)	14	147
DynB_K10 A	1.25 ± 0.07 (4)	12.6 ± 3.5 (4)	103 ± 14.2 (6)	10	82
DynB_G3A/Q8A	0.54 ± 0.13 (4)	86.1 ± 7.0 (4)	417 ± 52.0 (7)*	161	778
DynB_G3A/K10 A	1.57 ± 0.30 (4)	90.4 ± 7.6 (5)	497 ± 105 (7)*	58	317
DynB_Y1F	37.25 ± 6.27 (4) *	217 ± 13.5 (4)**	1,624 ± 534 (4)***	9	44
DynB_G3M	0.61 ± 0.04 (4)	117 ± 6.9 (4)*	85.3 ± 16.4 (4)	193	141
DynB_L5S	0.14 ± 0.02 (4)	18.6 ± 1.3 (4)	573 ± 22.6 (4)**	135	4,153
DynB_G3A/L5S	1.73 ± 0.58 (5)	98.3 ± 5.9 (4)	3,789 ± 366 (4)***	57	2,195
DynB_G3A/Q8H	0.30 ± 0.07 (4)	41.9 ± 1.3 (4)	460 ± 44.2 (4)*	137	1,508
DynB_Q8H	0.21 ± 0.06 (4)	7.7 ± 1.4 (5)	106 ± 17.1 (4)	36	507
DynB_G3M/L5S	7.24 ± 0.93 (4)	290 ± 17.9 (4)***	8,076 ± 890 (4)****	40	1,115
DynB_G3M/Q8H	0.36 ± 0.08 (4)	97.1 ± 15.8 (4)	167 ± 19.0 (4)	268	459

DynB variants with at least 10-fold increased selectivity for hKOPr over the hMOPr and/or hDOPr compared to wild-type DynB are highlighted in yellow. Statistical analysis was performed using the Kruskal–Wallis test with Dunn’s multiple comparison test; significance compared to DynB: *p < 0.05, **p < 0.01, ***p < 0.001, and ****p < 0.0001.

To identify promising candidates for enhanced gene vector-delivered Dyn effects, we aimed to select DynB variants with at least 10-fold increased selectivity for hKOPr over the hMOPr and/or hDOPr compared to wild-type DynB (DynB-WT). Among the DynB variants tested, seven fulfilled these criteria (see highlighted candidates in Table 1).

The selectivity over hMOPr/hKOPr of these seven DynB derivatives was at least comparable to that of DynA (DynB_G3A/L5S and DynB_G3M/L5S) or 3–5-fold higher (DynB_L5S < DynB_G3A/Q8H < DynB_G3A/Q8A < DynB_G3M < DynB_G3M/Q8H). The highest selectivity over hMOPr was obtained with DynB_G3M/Q8H. This variant showed a selectivity of 268-fold for hKOPr compared to the selectivity of DynB_WT of only 16-fold. Most of these variants also showed improved selectivity over hDOPr, the best exceeding the level of DynA_WT by 5-fold (DynB_G3A/Q8A < DynB_G3M/L5S < DynB_G3A/Q8H < DynB_G3A/L5S < DynB_L5S). The highest selectivity over hDOPr was shown by DynB_L5S. Substitution of the leucine residue with serine yields a 37-fold increased selectivity for hKOPr over hDOPr.

### G-protein activation (GTPγS)

Improved binding to the target receptor is not synonymous with higher receptor activation. Thus, to investigate how well the DynB variants activated the G-protein-coupled pathway of hKOPr, we performed GTPγS experiments, the gold-standard method to assess ligand potency and efficacy at defined target receptors. The endogenous ligand DynA is a full agonist with the ability to stimulate KOPr as efficiently as synthetic ligands, such as U50488 or U69593. Therefore, DynA was added to each experiment as a reference ligand, and the results from all the other peptides were normalized to those of DynA.

DynA proved to be approximately 10-fold more potent than DynB with an EC_50_ value of 0.60 ± 0.05 nM. Interestingly, DynB had a significantly (20%) higher E_max_ than DynA. The differences between DynA and DynB had been described as less pronounced in an earlier study (Chen et al., 2007).

None of the 13 DynB variant peptides displayed improved potency for hKOPr as compared to DynB. Three variants even displayed strongly reduced potency (Y1F, G3A/L5S, and G3M/L5S), while G3A/Q8A displayed a minor reduction in the potency. Peptides with a relative efficacy (E_max_) of approximately 85/90% or higher were considered full agonists. Mutations G3M, G3A/Q8H, G3M/L5S, and G3M/Q8H yielded an E_max_ value below this threshold and, therefore, were categorized as partial agonists.

The RBA experiment (see highlighted peptides in Table 2) and the GTPγS data suggest that DynB_L5S and DynB_G3A/Q8A have the most favorable pharmacological profile to activate the G-protein signaling cascade and being selective for hKOPr. This takes into consideration the fact that DynB_L5S and DynB_G3A/Q8A maintain a potency barely different from that of DynB_WT and still fully activate the receptor and the G-protein signaling cascade.

**TABLE 2:** Functional activities at the human kappa opioid receptor.

	Potency at hKOPr	Efficacy at hKOPr	n
	EC_50_ (nM ± SEM)	E_max_ (% ± SEM)	
DynA_WT	0.6 ± 0.05*	100.0 ± 0.0**	48
DynB_WT	5.10 ± 0.61	120.2 ± 1.5	4
DynB_G3A	27.86 ± 8.72	83.4 ± 5.5	4
DynB_Q8A	3.67 ± 0.52	125.1 ± 12.3	4
DynB_K10 A	9.79 ± 0.92	131.8 ± 3.9	4
DynB_G3A/Q8A	14.64 ± 1.57*	88.6 ± 3.1**	4
DynB_G3A/K10 A	16.68 ± 2.45	84.0 ± 3.6 **	4
DynB_Y1F	393.30 ± 47.28 *	110.4 ± 5.5	4
DynB_G3M	14.09 ± 4.17	54.5 ± 1.8****	4
DynB_L5S	17.74 ± 2.84	99.1 ± 2.1***	6
DynB_G3A/L5S	116.33 ± 11.20*	92.5 ± 5.7	4
DynB_G3A/Q8H	6.77 ± 0.82	74.9 ± 1.6****	4
DynB_Q8H	2.25 ± 0.47	111.5 ± 0.9 *	4
DynB_G3M/L5S	169.67 ± 23.67**	52.6 ± 1.6****	4
DynB_G3M/Q8H	5.43 ± 0.53	42.1 ± 1.3****	4

Highlighted in yellow are those DynB derivatives with 10-fold improved selectivity for hKOPr. Brown–Forsythe and Welch ANOVA; significance in comparison to DynB: *p < 0.05, **p < 0.01, ***p < 0.001, and ****p < 0.0001.

To compare the functional activities of DynB_L5S and DynB_G3A/Q8A to those of the other members of the opioid receptor family, we performed GTPγS experiments on hMOPr and hDOPr (see Table 3). Once again, DynA_WT displayed higher selectivity than DynB_WT for hKOPr, with 42-fold and 89-fold preference over hMOPr and hDOPr, respectively. This is in line with data published recently (Gomes et al., 2020). DynB_WT instead showed a lower degree of selectivity, with 6-fold and 12-fold preference over hMOPr and hDOPr, respectively (Table 3).

**TABLE 3:** Functional activities for DynB_L5S and DynB_G3A/Q8A at hKOPr, hMOPr, and hDOPr.

Stimulation of [35S]GTPγS binding											
	hKOPr			hMOPr			hDOPr			Selectivity	
	EC_50_ (nM ± SEM)	% stim (mean ± SEM)	n	EC_50_ (nM ± SEM)	% stim (mean ± SEM)	n	EC_50_ (nM ± SEM)	% stim (mean ± SEM)	n	MOPr/KOPr	DOPr/KOPr
DynA_WT	0.6 ± 0.0	100.0 ± 0.0	42	25.1 ± 7.2	100 ± 0.0	3	53.5 ± 10.0	99.9 ± 0.1	3	42	89
DynB_WT	5.1 ± 0.6	120 ± 1.5	4	31.4 ± 10.0	103 ± 0.0	3	59.3 ± 6.7	104 ± 3.6	3	6	12
DynB_G3A/Q8A	14.6 ± 1.6	88.6 ± 3.1	4	297 ± 46.6	111 ± 7.7	3	1,230 ± 105	86.4 ± 10.3	3	20	84
DynB_L5S	17.7 ± 2.8	99.1 ± 2.1	6	107 ± 8.6	93.4 ± 5.6	3	310 ± 50.4	86.7 ± 9.2	3	6	17

**FIGURE 1:**
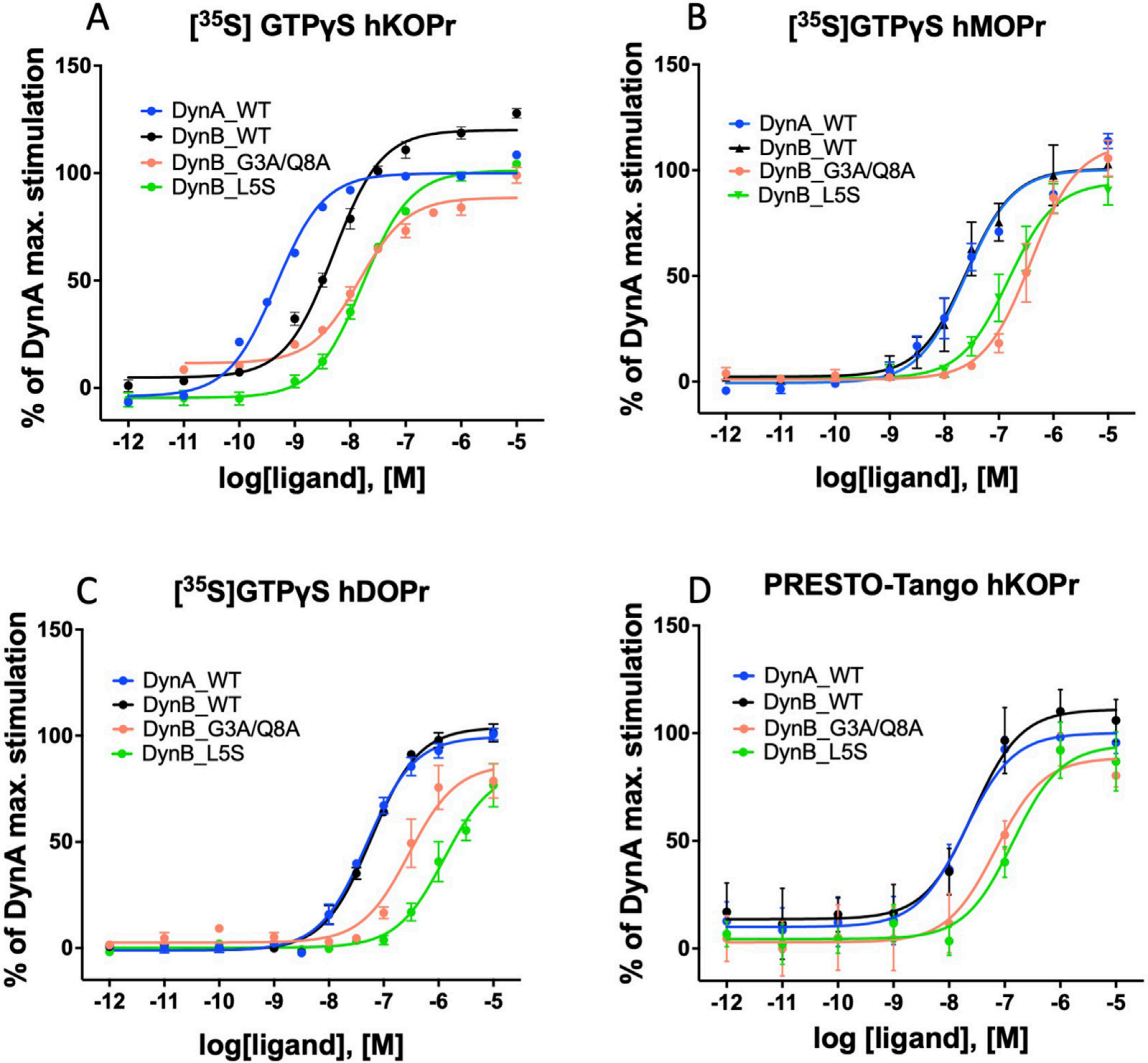
Activation of G-protein and β-arrestin by WT and modified dynorphins: dose–response curves for DynA, DynB, DynB_L5S, or DynB_G3A/Q8A on hKOPr **(A)**, hMOPr **(B)**, and hDOPr **(C)** in the GTPγS assay are depicted. Dose–response curves for DynA, DynB, DynB _L5S, or DynB_G3A/Q8A on hKOPr in the PRESTO-Tango assay are depicted in **(D)**. Data represent the mean ± SEM normalized to DynA.

DynB_L5S and DynB_G3A/Q8A displayed levels of selectivity for hKOPr similar or better than those for the wild-type peptide. Precisely, DynB_L5S selectivity over hMOPr was equivalent to that of DynB_WT, while selectivity over hDOPr was slightly improved (DynB_L5S = 17-fold vs DynB_WT = 12-fold). The double mutant DynB_G3A/Q8A, instead, displayed increased selectivity for hKOPr to 20-fold over hMOPr and 84-fold over hDOPr, taking its performance to the level of DynA_WT. Despite the 3-fold lower potency at hKOPr, the enhanced selectivity at the functional activity level for DynB_L5S and DynB_G3A/Q8A is due to a strongly reduced function on hMOPr and hDOPr (Figures 1A–C).

### β-arrestin recruitment (PRESTO-Tango)

β-arrestin recruitment triggers the G-protein-independent KOPr signaling cascade, which then acts as a scaffold protein or signal transducer of other signaling pathways. Thus, in order to understand whether DynB_L5S or DynB_G3A/Q8A possesses the characteristics of a biased agonist, it is necessary to evaluate their performance to recruit β-arrestin. To obtain these data, we performed PRESTO-Tango assays on cells overexpressing hKOPr. DynA_WT was used as the reference compound in each experiment.

DynA_WT exhibited the highest potency for β-arrestin recruitment. DynB_WT, instead, showed a non-significant tendency to higher EC_50_ values (Figure 1D). DynB_L5S displayed a significantly lower potency than DynB_WT. Although all tested DynB variants would be considered full agonists regarding β-arrestin recruitment, DynB_G3A/Q8A showed a lower stimulation of the receptor in comparison to DynB_WT (Table 4).

**TABLE 4:** β-Arrestin recruitment for DynA_WT, DynB_WT, DynB_L5S, and DynB_G3A/Q8A at hKOPr.

	Potency at hKOPr	Efficacy at hKOPr	n
	EC_50_ (nM)	E_max_ (%)	
DynA_WT	19.8 ± 2.1	100.0 ± 0.0	5
DynB_WT	29.2 ± 8.9	111.3 ± 5.3	3
DynB_L5S	145.7 ± 26.5*	95.1 ± 7.7	3
DynB_G3A/Q8A	66.1 ± 1.6	88.7 ± 2.6*	4

Brown–Forsythe and Welch ANOVA; significance in comparison to DynB: *p < 0.05.

### Biased factor calculation

To confidently identify a G-protein-biased peptide of interest, it is essential to keep in mind that the responses we collected through *GTPγS* and PRESTO-Tango assay are not solely determined by drug signaling properties but also by the way the cell and different assays “perceive” the generation of this response. These different “perceptions” of the pharmacological stimulus may themselves be responsible for an imbalance among signals from different pathways. To avoid this, and to be able to correctly estimate whether a peptide is indeed biased, we applied the method proposed by Kenakin et al. (2012), that is based on the operational method proposed by Black and Leff (1983). This method takes into consideration receptor occupation by the agonist, communication of the pharmacological stimulus to the system, and the processing of the stimulus by the system that produces the response.

**FIGURE 2:**
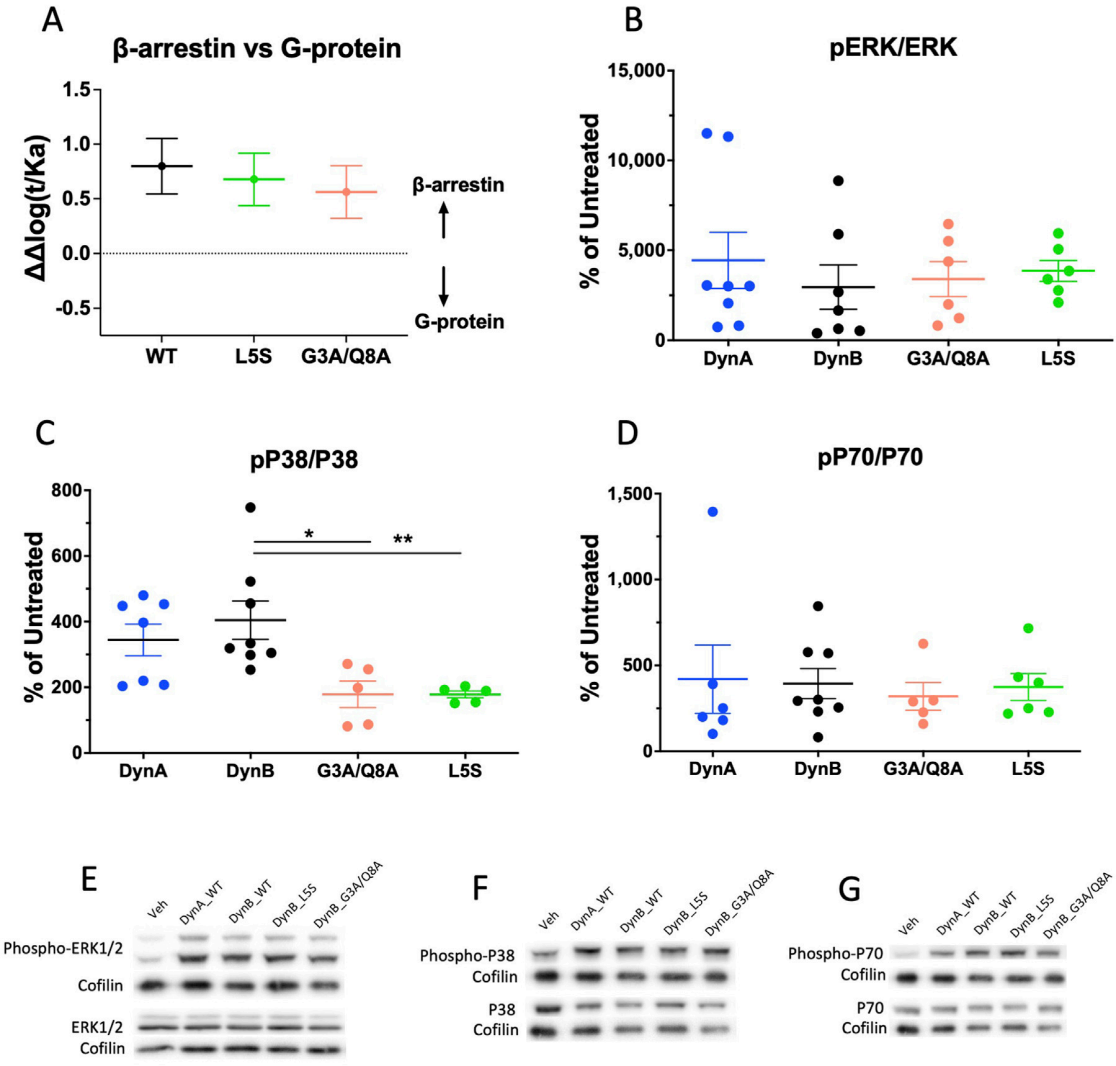
Bias plots for DynB_WT, DynB_L5S, and DynB_G3A/Q8A at hKOPr are depicted in **(A)**. Bias analysis was based on GTPγS and PRESTO-Tango assays. Data represent the mean ± SEM from three to six independent experiments. Western blot analysis of phosphorylation levels for hKOPr downstream targets after activation by DynA, DynB, DynB_G3A/Q8A, or DynB_L5S is shown in the other three panels. Quantitative analyses of the experiments are depicted for phospho-ERK 1/2 **(B)**, phospho-p38 **(C)**, and phospho-p70 **(D)**. Representative images of Western blots are depicted for pERK and ERK **(E)**, pP38 and P38 **(F)**, and pP70 and P70 **(G)**. Bars represent the mean ± SEM and are representative of five to eight independent experiments. *p < 0.05; **p < 0.01.

The analysis revealed that at hKOPr, DynB_WT has a biased factor of 0.8 ± 0.2 (Figure 2); thus, it is technically a β-arrestin-biased agonist compared to DynA. Similarly, DynB_L5S and DynB_G3A/Q8A exhibited β-arrestin bias, which did not differ from that of DynB_WT (bias factors of 0.7 ± 0.2 and 0.6 ± 0.2, respectively). These data indicate that DynB_WT is weakly biased for β-arrestin and that neither of the two selected DynB variants improve its pharmacological profile.

### Pathway analysis

It has been shown that mTOR pathway activation is necessary to induce KOPr-mediated aversion (Liu et al., 2018; Liu et al., 2019). However, the mechanism by which KOPr induces mTOR activity remains unclear; thus, it is impossible to say whether it is mediated by G-protein-, β-arrestin-, or by a third yet-to-be-discovered signaling cascade. To investigate whether our candidates activate mTOR to the same extent as wild-type Dyn, we performed Western blot experiments of phosphoproteins on cells stably transfected with hKOPr. In addition, this approach allowed us to examine biased agonism at the level of downstream signaling and, most importantly, of proteins directly associated with KOPr-mediated side effects (p38). Furthermore, phosphoprotein analysis was conducted in parallel and on the same samples for eliminating the intrinsic bias that is inevitable when using different assays. The proteins included in the experiments were ERK 1/2 (as a marker of G-protein activation), p38 (as a marker of β-arrestin activation), and p70 (as a marker of mTOR activation). Lysates of untreated cells of the same batch as treated cells were used as the reference.

Investigation of the phosphorylation levels for ERK 1/2 and p70 revealed no differences between DynB_WT and the two selected candidates. By contrast, p38 was significantly less phosphorylated by DynB_L5S and DynB_G3A/Q8A than by DynB_WT, which is in line with the tendencies observed in the PRESTO-Tango assays. Yet, this does not translate into less activation of the mTOR pathway. Therefore, the results from this experiment suggest that DynB_L5S and DynB_G3A/Q8A mostly retain the activation pattern for hKOPr downstream targets as DynB_WT (Figures 2B–D).

## Discussion

Our data demonstrate that modifications in positions 3, 5, and 8 of human DynB can increase the selectivity for hKOPr over hMOPr and hDOPr without any major impact on the affinity for hKOPr in binding assays. Mostly, the modification of glycine in position 3 to alanine, or methionine combined with the modification of leucine in position 5 to serine, or of glutamine in position 8 to alanine or histidine increased the selectivity for hKOPr. Our data expand the previous knowledge of the Dyn B function originating from alanine scan experiments (Joshi et al., 2017). Our data also indicate that the removal of a single hydroxyl group in position 1 (the Y1F modification) leads to a severe loss of affinity and potency. This underlined the importance of tyrosine in position 1 for the activation of hKOPr through interaction with the DQY motif in the ligand-binding pocket (Wang et al., 2023). Glycine in position 2 of DynA was reported to be important for KOPr selectivity and affinity. Substitution of this amino acid by D-alanine together with the substitution of glycine with des-glycine in position 3 led to a total loss of affinity to KOPr with only a minor impact on MOPr and DOPr binding (Guerrini et al., 1998). *In silico* and X-ray crystallography studies revealed that besides the Asp in position 138 of KOPr, which is conserved among all aminergic GPCRs and considered crucial for potency, intracellular loop 2 and extracellular loop 2 play important roles (White et al., 2015). The extracellular loop 2 interacts with amino acids C-terminally to the classical YGGF opioid motif. Indeed, the arginine residues in positions 6 and 7 were considered important for KOPr selectivity (Joshi et al., 2017). These two arginines form direct salt bridges with two glutamic acid residues in positions 209 and 297 of KOPr. Replacing either the arginine residues in dynorphin or the glutamic acid residues in the KOPr disrupts the dynorphin-induced activation of G_i_ by the KOPr (Wang et al., 2023). However, irrespective of the conserved binding affinity, the potency and efficacy of the modified peptides were mostly negatively affected. Thus, of all modified peptides with equal or improved affinity for hKOPr, only DynB_Q8H displayed comparable potency and slightly increased efficacy.

Taken together, our data indicate that higher peptide affinities do not necessarily result in higher potency or efficacy. This highlights the dual role of agonists to bind and activate the receptor. Conformational changes within the receptor are responsible for the recruitment to either G-proteins or β-arrestin (Mitra et al., 2021). A potential explanation for the discrepancies among affinity, potency, and efficacy is the involvement of distinct amino acids in ligand binding and receptor activation. Moreover, the combined interaction of multiple amino acids within DynB has to be considered. Likewise, the modifications in DynB_G3A/L5S and DynB_G3A/Q8H do not display significant changes in affinity compared to DynB_WT. However, potency decreases by more than 20-fold for DynB_G3A/L5S, but not for DynB_G3A/Q8H. Another interesting fact is that DynB_L5A displayed a minor reduction in affinity, but a marked increase in EC_50_ in the GTPγS assay (Joshi et al., 2017), while DynB_L5S did not display reduced potency (this study). This finding suggests that position 5 is important for the conformational shift of the receptor to induce signaling but is less involved in binding strength.

Despite increased selectivity for hKOPr, functional selectivity for the G-protein pathway would be an interesting add-on. Anticonvulsant effects of KOPr activation depend on presynaptic reduction of Ca^++^ influx, reducing the release of glutamate from excitatory cells and postsynaptic opening of K^+^ channels, inducing hyperpolarization. Both effects are mediated by G-proteins. Recruitment of β-arrestin and subsequent phosphorylation of KOPr trigger internalization and may reduce the anticonvulsant effects of KOPr agonists. Functional selectivity was described for a number of receptors, mostly for small-molecule drugs. Examples of peptides like exedin 4 analogs acting on GLP-1 or the cytokine CCL19 acting on CCR7 are less frequent (Madariaga-Mazón et al., 2017). Lohman et al. (2023) demonstrated that chemical modification of DynA could influence the phosphorylation of ERK, suggesting alterations in downstream signaling. Activation of the mTOR pathway considered responsible for adverse effects (Liu et al., 2018; Liu et al., 2019) was not studied. LOR17, a cyclic tetrapeptide (c[Phe-Gly-(β-Ala)-D-Trp]) demonstrated a strong G-protein bias at the hKOPr, providing analgesic effects without altering motor coordination, locomotor, and exploratory activities nor induced pro-depressant-like behavior (Bedini et al., 2020). Due to the use of artificial amino acids, LOR17 is not suitable for gene therapy. As our prime candidates chosen for their profile in selectivity, potency, and efficacy of hKOPr activation did not show a preference for the G-protein pathway over β-arrestin or mTOR activation, it remains open whether G-protein-biased peptidergic agonists can be designed.

## Data Availability

The original contributions presented in the study are included in the article/Supplementary Material; further inquiries can be directed to the corresponding author.
